# Study on the hydrothermal liquefaction of antibiotic residues with molecular sieve catalysts in the ethanol–water system: focus on product distribution and characterization†

**DOI:** 10.1039/d1ra03860e

**Published:** 2021-08-05

**Authors:** Jian Yang, Chen Hong, Yi Xing, Zixuan Zheng, Zaixing Li, Xiumei Zhao, Yongtao Lü, Jianwei Lü

**Affiliations:** Beijing Key Laboratory of Resource-oriented Treatment of Industrial Pollutants, University of Science and Technology Beijing Beijing 100083 China hongchen@ustb.edu.cn xing_bkd@163.com +86 15210342737 +86 13910550761; Department of Environmental Engineering, Hebei University of Science and Technology Shijiazhuang 050018 China 13832111831@163.com +86 13832111831; North China Pharmaceutical Co., Ltd. Shijiazhuang 050015 China

## Abstract

In this study, the antibiotic residue was used as a raw material to catalyze hydrothermal liquefaction (HTL) in an ethanol–water system to prepare bio-oil. The study explored the effects of ethanol–water ratio and three kinds of molecular sieve catalysts (HZSM-5, MCM-41, and γ-Al_2_O_3_) on the yield and characterization of bio-oil. The experimental results showed that the highest bio-oil yield was obtained at the ethanol–water ratio of 1 : 1 and the three kinds of molecular sieve catalysts of 15%. GC-MS, ^1^H NMR, TGA, and CHNS were used for the characterization of bio-oil. Higher carbon (up to 71.44%), hydrogen (up to 9.376%), and a high heating value (HHV, 34.714 MJ kg^−1^) were observed for catalytically liquefied bio-oil compared to non-catalytically liquefied bio-oil. The analysis of aqueous phase products indicated the existence of valuable nutrients. Besides, the reusability of three kinds of molecular sieve catalysts indicated that catalysts could be successfully reused several times and continuously exhibited the catalyst effect.

## Abstract 2

In this study, the antibiotic residue was used as a raw material to catalyze hydrothermal liquefaction (HTL) in an ethanol–water system to prepare bio-oil.

## Graphical abstract

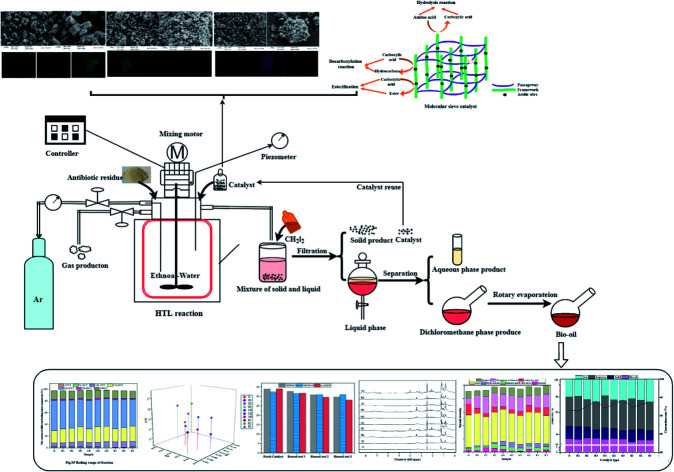

## 1 Introduction

Under the background that many countries around the world are advocating carbon neutrality, more and more scholars focus their research on biomass energy. Reducing the use of fossil energy and increasing the preparation of biomass energy is one of the most favorable means to reduce carbon emissions and achieve carbon neutrality as soon as possible.^1^

Biological waste is one of the sources of biomass energy. Using biological waste as the raw material for the preparation of biomass energy can not only dispose of biological waste harmlessly but also produce clean energy.^2^ The antibiotic residue is a by-product of the process of antibiotic preparation. For every ton of antibiotics produced, there will be 10 tons of antibiotic residue discharged into the environment, so the output is very huge. Emissions of antibiotic residue in China exceed 10 million tons per year.^3^ The antibiotic residue has a high viscosity, and its content of moisture is 79–93%. It is easy to deteriorate after long-term storage. It contains antibiotic residues and organic solvents, which are harmful to the ecological environment and human health, and it is one of the hazardous wastes in China. As China has prohibited the use of composting and the landfill treatment of antibiotic residue for a long time, the current treatment of antibiotic residue is mainly incineration.^4^ However, a large number of dioxins and other pollutants may be produced during the incineration process, which would cause serious pollution to the environment. Therefore, it puts forward high requirements for the flue gas purification of the incineration of antibiotic residue, which makes the disposal cost very high. A new approach to disposal is urgently needed.^5^

The basic principle of HTL is to heat antibiotic residues with water or other reaction solvents in a closed container.^6^ When the temperature and pressure rise to a certain extent, the solvent in the closed container will reach the sub/supercritical state.^7,8^ In this state, the reaction solvent will ionize hydrogen as a catalyst and hydrogen donor, which can promote decomposition and recombination reactions in antibiotic residue under high temperature and high pressure.^3^ Thus, bio-oil containing esters, olefins, ketones and other substances are produced.^9^ Since water is the reactant in this reaction, it is not necessary to reduce the moisture content, which makes the high moisture content in the antibiotic residue to be advantageous. In addition, according to Gong's research, the active site of antibiotics can be quickly inactivated at 90–180 °C.^10^ Thus, this method has good adaptability for the treatment of antibiotic residue.

During the process of hydrothermal liquefaction experiment, the use of water as a single solvent also has some disadvantages that cannot be ignored.^11^ First of all, the critical temperature of the water is relatively high, the specific heat capacity is also large, and the energy consumption to reach the critical state is relatively too much. Besides, bio-oil liquefied by water as a single solvent has high nitrogen and oxygen content and poor stability, which limits its use as an alternative fuel.^12^ Therefore, Peng proposed to add ethanol in water as a co-solvent for the HTL experiment. The results showed that the co-solvent can reduce the temperature and pressure of the reaction system, which is conducive to improving the stability and safety of the experiment.^13^ At the same time, the mixed solvent can also improve the hydrogen supply capacity of the solvent to increase the yield of bio-oil, and reduce the oxygen content of bio-oil, and finally improve the quality of bio-oil.^14^

Some researches focus on the addition of catalysts, which can be divided into homogeneous catalysts and heterogeneous catalysts. The homogeneous catalysts are mainly alkali metal salts such as Na_2_CO_3_ or KOH. These catalysts can improve not only the yield of bio-oil but also the quality of bio-oil.^15,16^ Zou found that the bio-oil yield increased to 25.8% when 5% Na_2_CO_3_ was added during the HTL of *Dunaliella salina*.^17^ Using Na_2_CO_3_ as a catalyst for HTL of microalgae. Burimsitthigul obtained bio-oil with a calorific value higher than that of crude oil.^18^ Every coin has two sides, and a homogeneous catalyst is no exception. The homogeneous catalyst is difficult to separate from the reaction system after the HTL reaction. If it is directly dumped, the homogeneous catalyst will cause pollution to the environment.^19^ The biggest advantage of heterogeneous catalysts is that they can be easily separated from liquefaction products and reused after proper treatment.^20^ Besides, molecular sieve catalysts also show good denitrification, deoxidation, and desulfurization activities, and their prices are low, so they are regarded as potential catalysts for the preparation of bio-oil by HTL.^21^ Savage used γ-Al_2_O_3_ molecular sieve catalyst to catalyze HTL of microalgae, and the results showed that the yield of bio-oil could be increased from 17 wt% to 30 wt%.^22^ Jin's study showed that HZSM-5 zeolite catalyst could improve the yield (40–54 wt%) and quality of bio-oil compared with other catalysts.^23^ These studies provided good references for the preparation of bio-oil by HTL of antibiotic residues. However, there are few studies on catalytic HTL in the organic reaction system. The question of whether there is a new reaction path of catalysts in the organic reaction system and what are the differences between HTL of antibiotic residues and algae needs to be further studied.

The effect of three kinds of molecular sieve catalysts (HZSM-5, MCM-41 and γ-Al_2_O_3_) on HTL of antibiotic residues were investigated. The effects of different catalysts and addition amounts on the yield of bio-oil were studied, and the bio-oil was analyzed by CHNS elemental analysis, TG analysis, GC-MS, and ^1^H NMR. In addition, nutrients (containing total nitrogen (TN), total organic carbon (TOC), ammonia nitrogen (NH_3_–N), and pH) in aqueous phase products were also analyzed.

These results could not only provide an environmentally friendly resource disposal method of antibiotic residue but also open a new window of clean energy of bio-oil prepared by HTL.

## 2 Materials and methods

### 2.1 Raw materials

The antibiotic residue was taken from the North China pharmaceutical factory in Hebei province, China. Using mortar and pestle, the dried antibiotic residue was reduced to 40 mesh size. The chemical composition of the antibiotic residue is shown in Table S1.† Molecular sieve catalyst (HZSM-5/γ-Al_2_O_3_/MCM-41) were purchased from Tianjin Yuanli Chemical Co., Ltd. The properties of the three molecular sieve catalysts are shown in Table S2.† SEM characterization and mapping characterization of the catalyst are shown in Fig. S1–S3.† As can be seen from these figures, the molecular sieve catalyst itself has a porous structure. There are more pores on the surface of the molecular sieve and the distribution is more uniform. The molecular sieve catalyst itself is mainly composed of Al, Si and O elements.

### 2.2 Catalyst hydrothermal liquefaction

**Fig. 1:**
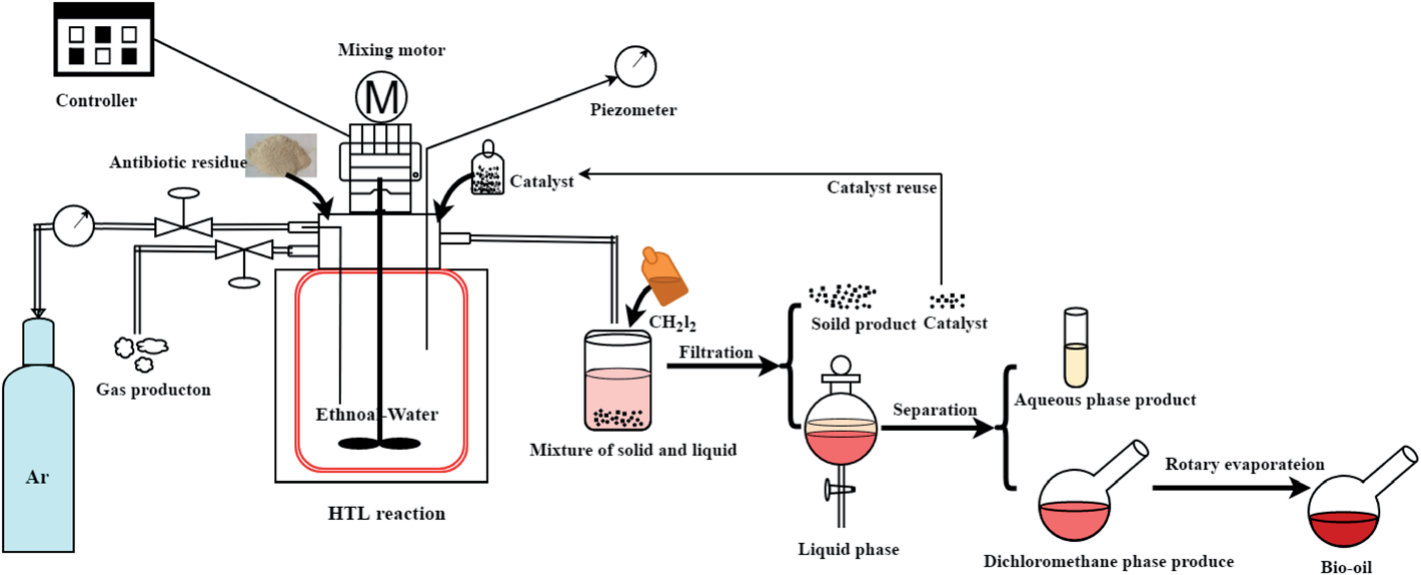
A products collecting route of catalyst hydrothermal liquefaction reaction.

The experiment operation and product collection method of the catalytic hydrothermal liquefaction reaction are shown in Fig. 1. First, antibiotic residue (18 g), the reaction solvent (60 ml of water and 60 ml of ethanol), and the catalyst were added to a 250 ml high temperature and pressure reactor. Argon gas was added to the reactor to replace the air, and the pressure was increased to 0.69 MPa before starting the reaction. After heating the reactor to 280 °C, the temperature was maintained and the reaction was continued for 150 min. During the reaction, the stirring speed of the reactor was maintained at 500 rpm. After that, the reactor was cooled and the gas was collected into the airbag. The solid–liquid mixture was washed with CH_2_Cl_2_ and filtered to obtain solid products (including catalyst) and liquid products. The aqueous and dichloromethane phase products were obtained by the extraction separation of dichloromethane and water. Bio-oil was obtained after the dichloromethane phase product was subjected to rotary evaporation and vacuum drying.^3^

Yields of bio-oil, solid product, aqueous phase product, and gas product were calculated as follows:1
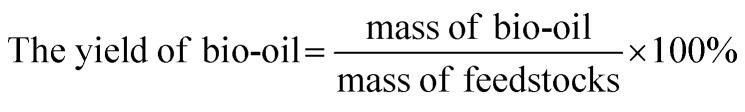
2
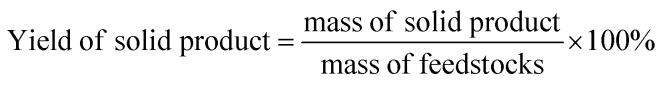
3
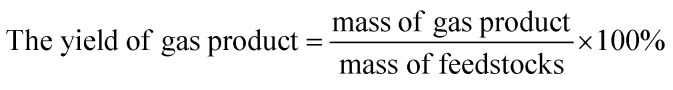

4 The yield of aqueous phase product = 100% − yield of gas product − yield of solid product − yield of bio-oil

5 Conversion rate = yield of the gas product + yield of aqueous phase product + yield of bio-oil

6 HHV (MJ kg^−1^) = 0.3516 × C + 1.16225 × H − 0.1109 × O + 0.0628 × N

All experimental results are averaged based on three parallel experiments.

### 2.3 Analyses methods

Elemental analyses (C, H, N, S/O) of the bio-oil were performed using a Vario EL 2400II element analyzer (PerkinElmer, USA). The O content was calculated by the difference method. ^1^H NMR spectra were obtained using a Bruker AV-600 spectrometer (Switzerland), and the solvent was deuterated acetone. GC-MS analysis was obtained using a GC-2010SE (Shimadzu. Japan), which was equipped with a DB-5MS UI chromatographic column. Compositions of the bio-oil were identified by the NIST spectrum library. The boiling range of bio-oil was obtained using a STA6000 TG analyzer (PerkinElmer, USA). Samples of bio-oil were heated from 20 °C to 700 °C at 10 °C min^−1^ in a nitrogen atmosphere. TN, TOC, N–NH_3_ in the aqueous phase of samples were obtained using a TNT832 (Hach, USA), Vario TOC (Elementar, German) and an N–NH_3_ reagent box (Fenke, China), respectively.

## 3 Results and discussion

In order to facilitate the discussion of bio-oil, the bio-oil samples were numbered in this study, with O, indicating that no catalyst was added, H10, H15 and H20 indicating the added amount of HZSM-5 as 10/15/20 wt%, M10, M15 and M20 as the added amount of MCM-41 as 10/15/20 wt%, R10, R15 and R20 as the added amount of γ-Al_2_O_3_ as 10/15/20 wt%, respectively.

### 3.1 Effect of catalysis on hydrothermal liquefaction product distribution

**Fig. 2:**
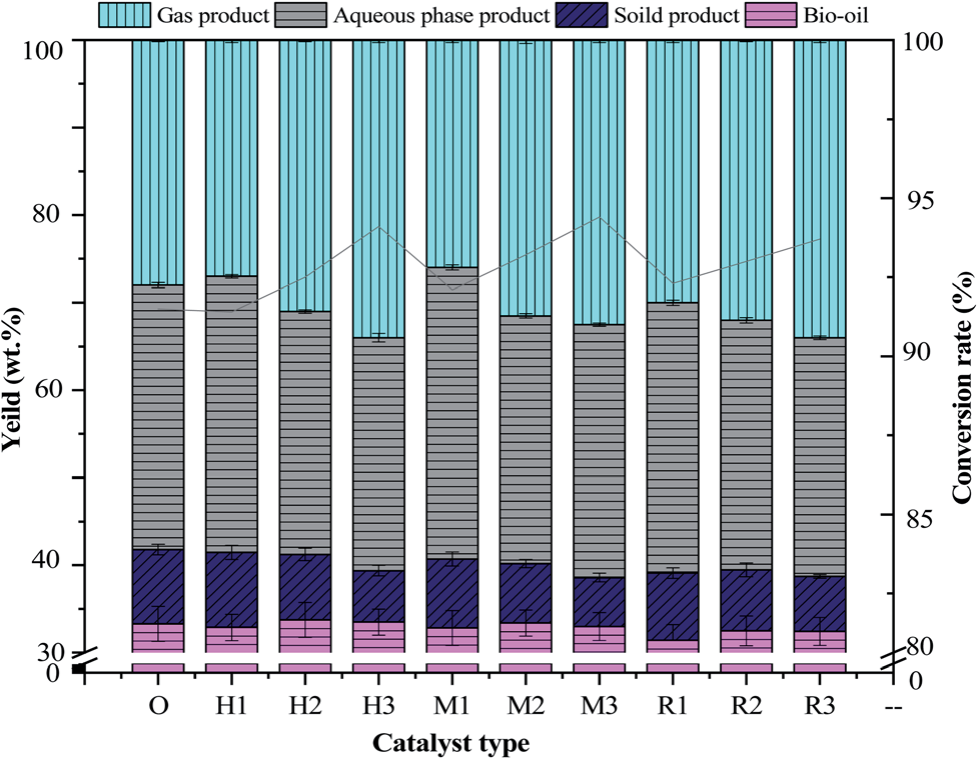
Effect of catalysis on hydrothermal liquefaction product distribution.

The effects of three kinds of molecular sieve (HZSM-5, MCM-41 and γ-Al_2_O_3_) with additions of 0%, 15%, 10%, and 20% on the products and conversion rates of antibiotic residue are shown in Fig. 2. When additions of HZSM-5 and MCM-41 and γ-Al_2_O_3_ are 15%, the corresponding yields of bio-oil reach the highest of 33.74 wt%, 33.38 wt% and 32.48 wt%, respectively. A comparison of non-catalytic bio-oil indicated that the influence of adding catalysts on the yield of bio-oil was not obvious. Although the addition of the catalyst can further promote the hydrothermal liquefaction reaction and thus improve the reaction progress.^24^ After catalysis, part of the heavy oil with more oxygen in the bio-oil will remove O and it turns into light oil, and the density of bio-oil will decrease, thus reducing the yield of bio-oil.^25^ The HTL of light oil increases, and the proportion of the weight of bio-oil reduces, which finally reduces the yield of bio-oil. Compared to non-catalysts, 20% addition of catalysts produces more gaseous products with the highest yields of 33.92 wt%, 32.46 wt% and 34.04 wt%. This is because more catalyst increases the active surface of the reactant and the hydrothermal cracking rate.^26^ When the additions of HZSM-5, MCM-41 and γ-Al_2_O_3_ are 10%, yields of solid products are 8.6%, 7.5% and 7.7%, respectively, which has little difference from that in the absence of the catalyst. With the increase in the addition of the three kinds of catalysts, the amount of solid products decreases and higher conversion rates are obtained.^26^ The highest conversion rates of 94.1%, 93.4% and 93.7% are achieved at 20% catalyst amounts of HZSM-5, MCM-41 and γ-Al_2_O_3_, respectively. This may be due to the fact that the presence of a small amount of catalyst promotion has a limited effect on the HTL of antibiotics residue.^27^ The maximum addition (20%) of the catalyst will lead to a more cracking reaction, meanwhile, some solid products will be re-converted in the HTL reaction, leading to a decline in the yield of solid products and an increase in conversion rate.^20,28,29^

### 3.2 Bio-oil analysis

#### 3.2.1 Elemental composition and HHV of bio-oil



7

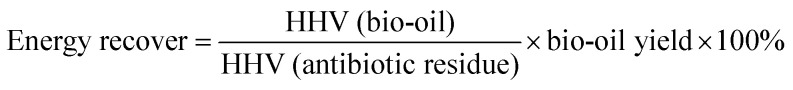

The elemental analysis of the bio-oil samples before and after adding the catalyst is shown in Table 1 and Fig. S5.† It can be seen from the table that the catalyst can reduce the O content in bio-oil and increase C, H and N contents in bio-oil. The O content in bio-oil without the catalyst was about 11.63%. After the addition of HZSM-5, the O content in bio-oil showed a straight downward trend with an increase in the amount of HZSM-5. When the amount of HZSM-5 reached 20%, the O content in bio-oil decreased to 8.66%, and H showed a slight upward trend, rising from 8.37% to the maximum of 9.38%. This is similar to Zhang's research result that the decarboxylation reaction in the reaction system was increased because of the addition of HZSM-5.^30^ When the amount of MCM-41 catalyst reached 20%, the O content decreased to 10.195%, and the H content increased to 8.44%. This was caused by the addition of MCM-41 catalyst in the HTL reaction, which promoted the intensification of deoxidation reaction in the reaction.^31^ After the addition of γ-Al_2_O_3_ reached 20%, O decreased to 8.67% and H increased to 9.41%, which was caused by the hydrogenation deoxidation mechanism of γ-Al_2_O_3_.^32^ Generally speaking, the higher H/C in the bio-oil, the lower O/C and N/C, the bio-oil could have a high quality. As can be seen from Fig. S5,† the Van Krevelen diagram, the bio-oil quality with the addition of the catalyst is significantly higher than that without the addition of catalyst, and the higher the amount of catalyst, the better is the quality of bio-oil. This is due to the removal of oxygen content from bio-oil through catalytic liquefaction. Bio-oil also shows a higher HHV with a higher bio-oil yield.^33,34^ As a result, the energy recovery rate of bio-oil after the addition of catalysts also increases. Meanwhile, the N content in bio-oil is also increased, which is similar to the results of Ma's study.^35^ This is mainly because of the fact that adding catalyst will lower HTL in the synthetic reaction activation energy, making partial N gaseous products stay in the bio-oil or aqueous phase in the product. At the same time, HZSM-5/MCM-41 and γ-Al_2_O_3_ three kinds of molecular sieve catalysts have a larger specific surface area, which would provide more catalytic reaction sites, thereby increasing the reaction in the raw material of nitrogenous substances converted into bio-oil.^35^ The O/C atomic ratio of the catalytic bio-oil is below 0.1, which is about 10% lower than the 0.12 of the non-catalytic bio-oil. This is similar to the results of Ramya^28^ and Yan's^19^ study. The addition of acidic catalysts can increase the solubility of molecular hydrogen in the reaction system, thus promoting the further occurrence of hydrogenation and deoxidation. Although the HHV of bio-oil is close to that of petroleum crude oil (O content is 1%, N content is 0.3%, HHV = 42 mJ kg^−1^), the nitrogen content of bio-oil is still much higher than that of crude oil. Therefore, bio-oil still needs further denitrification refining before it could be used in refineries.^9^

**Table 1:** Elemental composition

Sample	Elemental composition (%)					H/C	O/C	N/C	Empirical formula	HHV (MJ kg^−1^)	Energy recovery (%)
	C	H	N	S	O^a^						
O	70.29	8.367	8.937	0.777	11.629	1.428	0.124	0.109	CH_1.43_O_0.12_N_0.11_	32.588	54.33674
H1	70.92	8.635	9.687	0.788	9.97	1.461	0.105	0.117	CH_1.46_O_0.11_N_0.12_	33.257	54.75406
H2	71.44	8.91	9.567	0.802	9.281	1.497	0.097	0.115	CH_1.50_O_0.10_N_0.12_	33.844	57.19427
H3	71.29	9.376	9.874	0.802	8.658	1.577	0.091	0.119	CH_1.57_O_0.10_N_0.12_	34.372	57.63931
M1	69.77	7.991	9.132	0.801	12.306	1.374	0.132	0.112	CH_1.37_O_0.13_N_0.11_	31.880	52.39115
M2	69.98	8.597	9.614	0.741	11.068	1.474	0.119	0.118	CH_1.47_O_0.12_N_0.12_	32.766	54.78128
M3	71.33	8.442	9.227	0.806	10.195	1.420	0.107	0.110	CH_1.42_O_0.11_N_0.11_	33.181	54.81139
R1	70.52	8.403	8.674	0.845	11.558	1.430	0.123	0.105	CH_1.43_O_0.12_N_0.11_	32.735	51.48319
R2	71.08	8.711	9.032	0.801	10.376	1.471	0.109	0.109	CH_1.47_O_0.11_N_0.11_	33.398	54.3333
R3	71.71	9.51	9.413	0.699	8.668	1.591	0.091	0.112	CH_1.59_O_0.10_N_0.11_	34.714	56.3693

a Calculated by difference (100% − C% − H% − N% − S%).

#### 3.2.2 Chemical composition of bio-oil

**Fig. 3:**
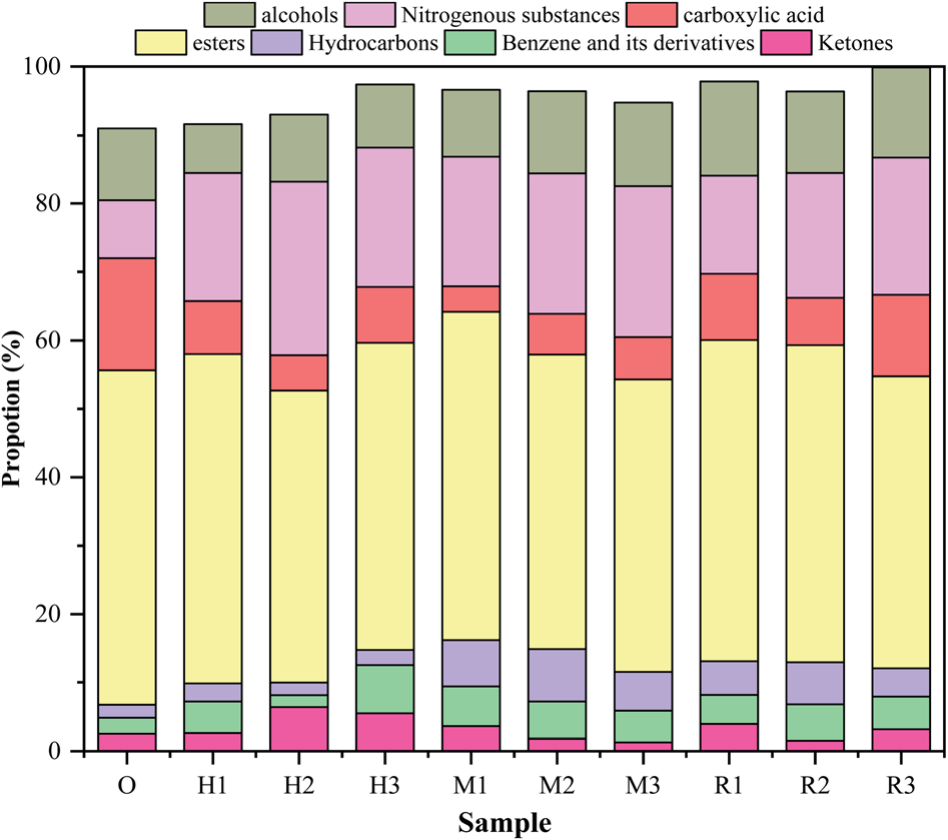
Effect of catalyst on the chemical composition of bio-oil.

Bio-oil prepared by HTL reaction is a complex system composed of a variety of oxygen and nitrogen organics. In this study, the organic matter in bio-oil is divided into hydrocarbon, alcohol, ester, carboxylic acid, nitrogen (amine, *etc.*) substances, benzene ring and derivatives, ketones, and others. The summary of the main components of bio-oil and chemical classification is shown in Fig. 3. The sum of the peak area of the main components is more than 90% of the total ion spectrum area.^3^

Most hydrocarbons in bio-oil come from the deacidification of fatty acids and the chain formation of aromatic substances.^36^ In this study, it was found that olefins were the only hydrocarbons formed in bio-oil when the reaction was performed without adding the catalyst, while hydrocarbons formed in the bio-oil after adding the catalyst included not only olefins but also alkanes. In our previous study, we found that 5,7,13,15-tetramethyl-2-tetradecane only appeared in the ethanol–water reaction system, but not in the pure water reaction system.^3^ When the addition of HZSM-5 was 20%, alkane 5,7,13,15-tetramethyl-2-tetradecane (residence time (RT) of 17.771) content of bio-oil reached up to 1.76%. This was caused by the strong acidity, high activity and shape-like properties of HZSM-5. When MCM-41 addition was 20%, the 5,7,13,15-tetramethyl-2-tetradecane (RT17.771) content was 0.96%. This is mainly due to MCM-41 belonging to mesoporous molecular sieves has a larger specific area, more conducive to the macromolecular depolymerization, aliphatic group and generated hydrocarbons^30^ than microporous molecular sieves. In addition, MCM-41 also shows a certain amount of acid sites leading to fatty acid reaction forming hydrocarbons, accompanied by a species of aromatic substances forming a open-loop chain.^37,40^ When the addition of γ-Al_2_O_3_ is 20%, the content of 5,7,13,15-tetramethyl-2-tetradecane (RT17.771) is 0.7%. This is because there are weak acid sites on the surface of the γ-Al_2_O_3_ catalyst, which can be used as active sites for the catalytic dehydration reaction.^38^ Thus it promotes the hydrogenation of bio-oil by the hydroxyl group of ethanol in the reaction system. The content of benzene and its derivatives in the bio-oil without adding catalyst was 2.35%. However, when the amount of HZSM-5 was 20% and the amount of MCM-41 was 10% and the amount of γ-Al_2_O_3_ was 15%, the corresponding content of benzene and its derivatives reaches 7.07%, 4.56% and 4.77%, respectively. This is because that HZSM-5 has an aromatization property and the more catalysts are added, the more benzene rings and derivatives are produced. The structure of MCM-41 has the property of ring-opening, and it will open the chain of aromatic compounds after adding too much MCM-41.^20^ The competitive mechanism between the strong acid and weak acid sites of γ-Al_2_O_3_ has little effect on benzene and its derivatives.^39^ The esters in bio-oil showed an increasing trend with the increasing addition of catalysts. It is because the three catalysts are acidic, which is conducive to the ethanol in the reaction system to participate in the esterification reaction and transesterification reaction, thus leading to the increase in the ester content in the bio-oil.^10^ The contents of carboxylic acid material and ketones tend to decrease after adding the catalyst. This trend is consistent with elemental analysis results, which is due to deacidification of these three kinds of catalysts.^20,40^ The nitrogenous substance content is 8.5% without adding catalysts. When the content of HZSM-5/MCM-41/γ-Al_2_O_3_ reaches 15%, 15% and 20%, respectively, the nitrogen contents of bio-oil reached up to 25.33%, 20.55% and 20.1%, respectively. This is similar to the results of studies from Ma,^35^ Cheng^41^ and Chen,^12^ which may be because the special pore structure of these three catalysts can provide more catalytic reaction sites, leading to more nitrogen-containing substances entering the bio-oil. At the same time, in the initial stage of HTL, the antibiotic residue can be hydrolyzed as sugars and amino acids.^12^ Maillard reaction can be carried out between sugars and amino acids to produce nitrogen-containing substances (pyridine, pyrrole, anthraquinone, and so on) in a high-temperature and high-pressure reactor.^12^ The addition of catalysts can further promote the Maillard reaction, thus increasing nitrogen content in the bio-oil.^24^

#### 3.2.3 Boiling range distribution

Thermogravimetric analysis (TGA) is similar to the distillation process of bio-oil from low to high temperatures. Although there may be a small amount of bio-oil pyrolyzed, the TGA results can still provide researchers with a general boiling range distribution.^3^

Fig. S6† shows the boiling range distribution of bio-oil samples under different reaction conditions. Table 2 shows the specific weight loss percentage. In the inert atmosphere, when the bio-oil was heated to 70 °C, the weight loss of bio-oil was less than 0.5%. It indicates that the volatile components in bio-oil had been effectively removed before the test. Under the same addition of three catalysts, the changing trends in weight loss of bio-oil are similar. It is because all these three kinds of catalysts are molecular-sized and acidic catalysts.^41^ There are certain similarities in the chemical composition of bio-oil produced by catalysis. In Peng's study, bio-oil components above 600 °C were converted into fixed carbon.^14^ Compared with bio-oil prepared without a catalyst, the ash content of bio-oil prepared by catalysis (17.17%) was significantly higher, indicating that the addition of catalysts can reduce the fixed carbon content of bio-oil. It can be seen from Fig. S6† and Table 2 that there are three weight loss intervals for bio-oil. Generally speaking, the weight loss interval for light oil, intermediate components and heavy oil are 120–170 °C, 170–250 °C and 250–500 °C, respectively.^42^ The catalytic HTL of antibiotic residue results in the increase of light oil and intermediate components. In the absence of catalysts, the preparation of bio-oil with antibiotic residue is mainly through hydrolysis, recombination, esterification, deamination, and other reactions, and only the synergy of ethanol–water is involved in the whole HTL process.^3,43^ Therefore, the transformation of major components of antibiotic residue may not be complete, and many macromolecular substances still exist in bio-oil. After the addition of catalysts, the catalytic properties of the catalyst itself (such as dehydrogenation of HZSM-5 catalyst, MCM-41 decarylation and γ-Al_2_O_3_ dehydration) will continue to convert the macromolecular substances in the antibiotic residue into smaller substances, thus further reducing the boiling range of the bio-oil.^43^ Compared with petroleum crude oil, the composition of bio-oil with a boiling point below 250 °C is similar to those of gasoline and diesel oil, which belong to the part that can be directly burned; in the 250–500 °C part, similar to the composition of lubricating oil, it belongs to the usable part; the part over 500 °C is the part that needs to be refined, which belongs to the part that cannot be used directly. On the whole, the compositions of the bio-oil directly or useable by using the catalyst were improved. It can also be indicated that the catalyst has a significant improvement in hydrothermal liquefaction to prepare bio-oil.

**Table 2:** Boiling range

Boiling range of fraction (% of each integral)										
boiling range (°C)	O	H1	H2	H3	M1	M2	M3	R1	R2	R3
<70	0.16	0.22	0.24	0.23	0.15	0.25	0.21	0.17	0.25	0.23
70–120	1.32	1.38	1.52	1.98	1.44	1.55	2.02	1.24	1.79	1.51
120–170	6.74	7.12	7.93	8.55	7.61	7.88	8.67	9.32	9.44	9.52
170–250	21.07	23.33	24.15	25.66	22.92	26.17	26.06	21.71	22.08	26.63
250–500	52.01	49.03	48.05	46.9	48.86	47.1	47.21	48.62	48.71	45.41
500–600	1.53	2.14	1.61	1.45	1.51	2.02	1.54	1.57	1.32	1.15
>600	17.17	16.78	16.5	15.23	17.51	15.03	14.29	17.37	16.41	15.55

#### 3.2.4 The functional groups of bio-oil

**Fig. 4:**
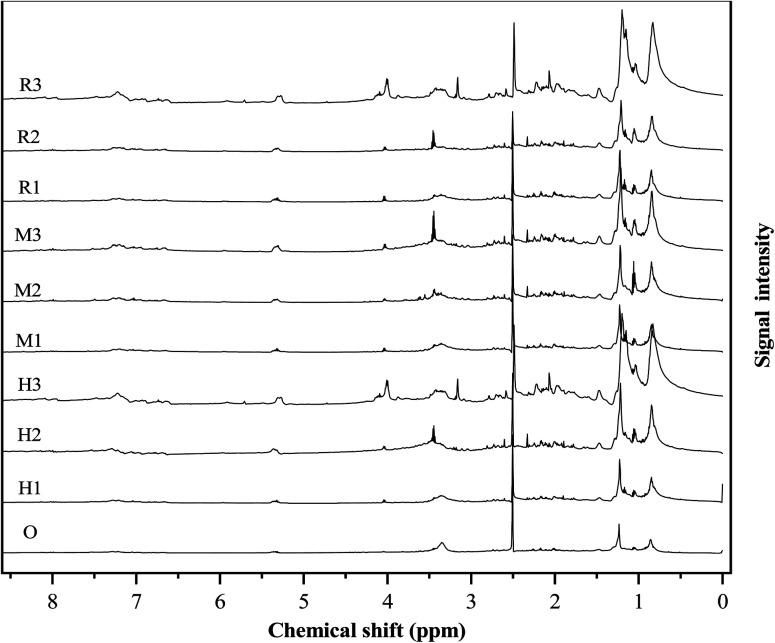
^1^H NMR spectra of bio-oils.

As can be seen, from Fig. 4, the major influence of the catalyst on bio-oil is reflected in the region of 0.5–1.5, 1.5–4.0, 3.0–4.4, 4.4–6.0 and 6.0–8.5. The corresponding region areas are listed in Table S4.†

In the ethanol–water reaction system without a catalyst, the ester proton region of 0.5–1.5 ppm shows the highest percentage of 61.42%. After adding the catalyst, the ester sub-region area shows a trend of rising. The ester proton region area reaches up to 84.7% at γ-Al_2_O_3_ content of 20%, which is consistent with the results of GC-MS. This is because the three kinds of catalysts are acidic and promote HTL reactions of esterification and ester exchange.^35^ The region of 1.5–3.0 ppm reflects unsaturated bond and hydrogen alpha heteroatoms. Compared with the addition of the catalyst, more protons are observed in the bio-oil without catalyst. It can be seen that three kinds of molecular sieve catalysts have a direct catalytic effect.^41^ The 3.0–4.4 ppm region corresponds to hydroxyl and ether-bonded hydrogen. In all bio-oil samples, the percentage of protons in this region is very small, only 0.71–1.33%. The results of this study are different from those of Peng's, who reported that the protons in bio-oil were about 10% in a region of 3.0–4.4 ppm.^14^ It can be seen from Table S4† that antibiotic residue contains much less carbohydrate and ester substances than algae, so more ether bonds, and hydroxyl groups are needed to provide free radicals.^44^ The region of 4.4–6.0 ppm corresponds to the hydrogen of the methoxyl group, showing that there are a large number of hydrogen atoms containing aromatic ethers protons and carbohydrate molecules. These substances are intermediate products in the hydrothermal liquefaction process. The protons in this region decrease with increasing catalyst content, which is consistent with the results of the elemental analysis. This is due to the addition of the catalyst to make the HTL reaction more thorough, the increase of final products, thus reducing the intermediate products.^45^ The region of 6.0–8.5 ppm corresponds to aromatics, whose content increases with the addition of HZSM-5 but decreases with the addition of MCM-41. This trend is consistent with the GC-MS results. This is due to the aromatization of bio-oil induced by HZSM-5 and the open-loop performance of MCM-41.^20^

### 3.3 Aqueous phase analysis

According to Ma's study,^35^ the aqueous phase products contain valuable nutrients (such as NH_3_–N, TOC, TN, and pH), so we analyzed the nutrients in the aqueous phase products.

The TN, TOC, NH_3_–N, and pH of aqueous phase products obtained from the catalytic HTL and non-catalytic are shown in Table 3. Compared with the catalytic aqueous phase products (2866–3715 mg L^−1^), the content of NH_3_–N in the non-catalytic liquid aqueous phase products is as high as 3878 mg L^−1^. It can be seen from the table that these three molecular sieve catalysts have a certain influence on the content of NH_3_–N in aqueous phase products.^21^ The increase in the amount of catalyst can reduce the content of NH_3_–N in aqueous phase products. When the addition of HZSM-5, MCM-41, and γ-Al_2_O_3_ sieves reaches 20%, the NH_3_–N content of aqueous phase products decreases to 3008, 2997 and 2886 mg L^−1^, respectively. The result is consistent with that of element analysis, which may be due to the increase of conversion of amino acids under the action of the catalyst.^24^ The catalytic liquid aqueous phase can significantly reduce the TN content, which is consistent with the results of GC-MS. It may be due to the further conversion of some water-soluble amino acids into bio-oil under the catalysis of molecular sieve, resulting in the decrease of TN in the aqueous phase products.^11^ The TOC (19 772–28 966 mg L^−1^) of the catalytic aqueous phase product is much lower than that of the non-catalytic (30 132 mg L^−1^). With the increase in the amount of catalyst, the TOC in the aqueous phase product shows a trend of linear decline. This result is close to Ma's study, in which, it was reported that the molecular sieve catalyst promoted the HTL reaction.^34^ Compared with non-catalyst (pH = 8.7), the pH (5.8–8.0) of the catalytic aqueous phase product was reduced. This is because the three molecular sieve catalysts are all acidic, and the pH of the solvent will be reduced when the molecular sieve enters the ethanol–water reaction system. At the same time, the addition of the catalyst leads to the generation of some water-soluble organic acids in the hydrothermal liquefaction reaction process, thus reducing the pH value of the aqueous phase products.^6,46^ On the whole, the nutrients in the aqueous phase products after the addition of catalysts showed a decreasing trend. It can be seen from the side that the addition of three molecular sieve catalysts can significantly reduce the loss of nutrients into the aqueous phase products and improve the actual production efficiency of HTL.

**Table 3:** Distribution of nutrients in aqueous phase products

Sample	NH_3_–N (mg L^−1^)	TN (mg L^−1^)	TOC (mg L^−1^)	pH
O	3878	8768	30 132	8.7
H1	3715	6951	27 045	7.2
H2	3361	5817	22 914	6.5
H3	3008	4984	19 772	5.8
M1	3477	6233	28 610	8.0
M2	3244	4975	25 008	7.4
M3	2997	4032	22 434	6.8
R1	3362	7358	28 966	7.1
R2	3153	6424	24 617	6.0
R3	2866	5546	20 305	5.5

### 3.4 Catalyst reusability analysis

**Fig. 5:**
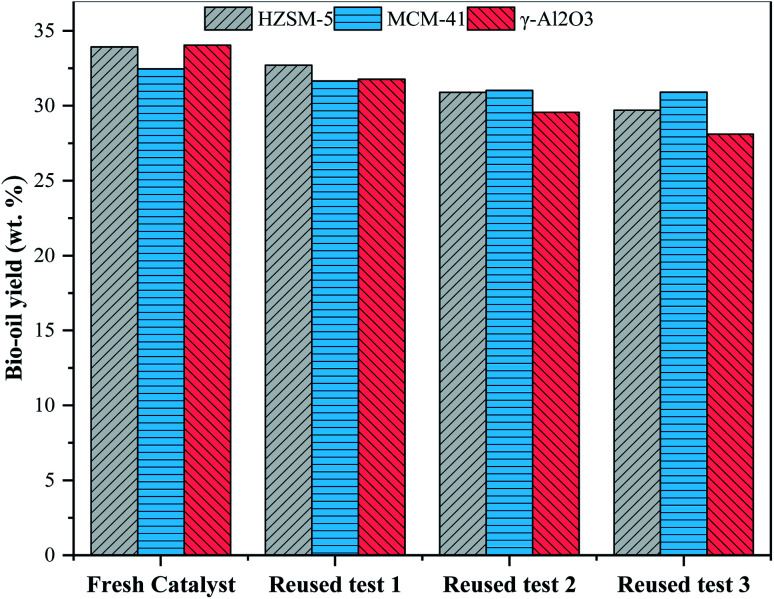
Effect of reuse of zeolite catalyst on bio-oil yield.

The reusability of three kinds of molecular sieve catalysts with an addition of 15% was studied. Three kinds of molecular sieve catalysts were recovered from the mixture of solid product and catalyst through calcination in a muffle furnace in the presence of oxygen at 550 °C for 5 hours. The yield of bio-oil recovered with the catalyst is shown in Fig. 5. The results show that the HZSM-5 catalyst recycling has a certain influence on the yield of bio-oil. When the catalyst is recycled for the third time, the yield of bio-oil drops to 29.7 wt%. It is because HZSM-5 possesses inadequate activity, resulting from the slightly reduced acidity and blocked surface-active site after a few recycling times.^21,47^ The recovery of MCM-41 catalyst has the least influence on the yield of bio-oil, which may be due to that MCM-41 has a larger pore diameter and is not easily blocked.^28^ Recycling of γ-Al_2_O_3_ catalyst affects the yield of bio-oil most significantly. After the third recycling, the yield of bio-oil dropped to 28.11 wt%. This may be because the pores on the surface of the catalyst were blocked by inorganic ash or carbon and thus reduced its activity. Another reason may be that the γ-Al_2_O_3_ has been reduced to Al^2+^ in the process of HTL, and Al^2+^ falls off from the molecular sieve and is retained in bio-oil or aqueous phase products.^39^ The above results confirm that three kinds of molecular sieve catalysts have certain potential reusability in the preparation of bio-oil by catalytic HTL of antibiotic residues in the ethanol–water system, with MCM-41 presenting the best reusability.

## 4 Conclusion

The effect of molecular sieve catalysts on the preparation of bio-oil by HTL of antibiotic residue in the ethanol–water reaction system was studied. Results showed that the quality of bio-oil after catalysis has been improved, and the yield has not been improved. The elemental analysis results showed that H was increased (up to 9.51%), O was slightly decreased (down to 8.668%), and the high calorific value (34.71 MJ kg^−1^) and energy recovery (up to 57.64%) of bio-oil were increased. From the point of view of the composition of bio-oil materials, hydrocarbon substances also show a trend of increase with the addition of catalysts. The boiling range of bio-oil after the addition of catalyst also shows a trend of decrease, which means that the carbon chain of bio-oil is shortened, and the decrease of nutrient content in the aqueous phase products also proves the positive catalytic effect of catalyst from the side. In this study, cheap molecular sieves were added as catalysts instead of precious metal catalysts, in order to provide a possibility for practical production. In addition, it is also found that the catalysts have a certain reuse value, which can provide the possibility of further reducing industrial costs in actual production.

## Data availability

Data will be made available on request.

## Author contributions

Jian Yang: conceptualization, methodology, software. Priya Singh: data curation, writing-original draft preparation. Chen Hong and Yi Xing: visualization, investigation, supervision. Zixuan Zheng: writing – reviewing and editing. Zaixing Li and Xiumei Zhao: software, validation. Yongtao Lü and Jianwei Lü: funding acquisition. All authors reviewed the manuscript.

## Conflicts of interest

The authors declare that they have no known competing financial interests or personal relationships that could have appeared to influence the work reported in this paper.

## Supplementary Material

RA-011-D1RA03860E-s001
