# Supplementary material for: Study on the hydrothermal liquefaction of antibiotic residues with molecular sieve catalysts in the ethanol–water system: focus on product distribution and characterization
Source: RSC Adv. 2021 Aug 5;11(43):26763–72. doi: 10.1039/d1ra03860e (PMC9037444; doi:10.1039/d1ra03860e)
Supplement: RA-011-D1RA03860E-s001 [file RA-011-D1RA03860E-s001.pdf]

Electronic Supplementary Material (ESI) for RSC Advances.
This journal is © The Royal Society of Chemistry 2021

Table S1 Characteristics of antibiotic residue

	Parameter	Content
Proximate analysis	Volatile matter (wt%)	75.26
	Fixed carbon (wt%)	8.51
	Ash (wt%)	7.32
	Moisture(wt%)	8.91
Ultimate analysis	C (%)	47.38
	H (%)	6.62
	N (%)	6.26
	S(%)	0.81
	O[①] (%)	36.02

① Calculated by difference (100%-C%-H%-N%-S%-Ash%).

Table S2 Properties of catalysts

Catalyst Type	Surface area, m^2/g	Pore diameter, A^0
HZSM-5	380	5

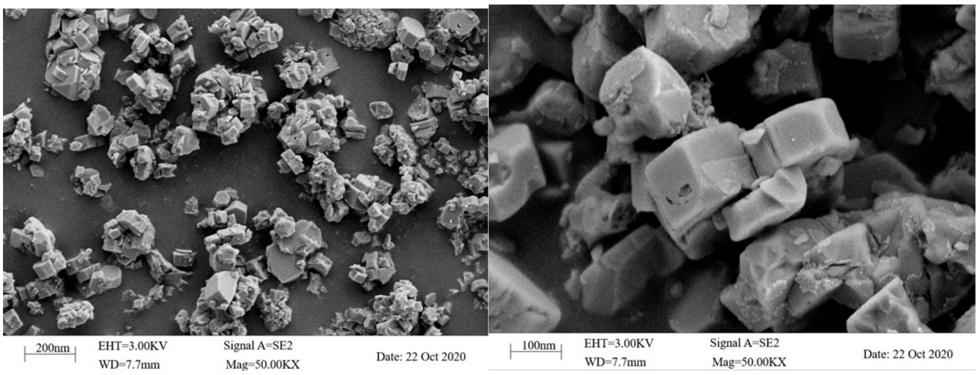

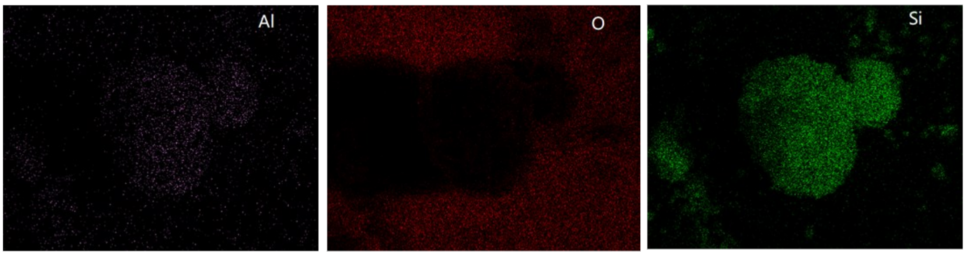

MCM-41	1000	3.6
γ-Al_2O_3	320	12

Fig. S1 Characterization of HZSM-5 molecular sieve by SEM and mapping

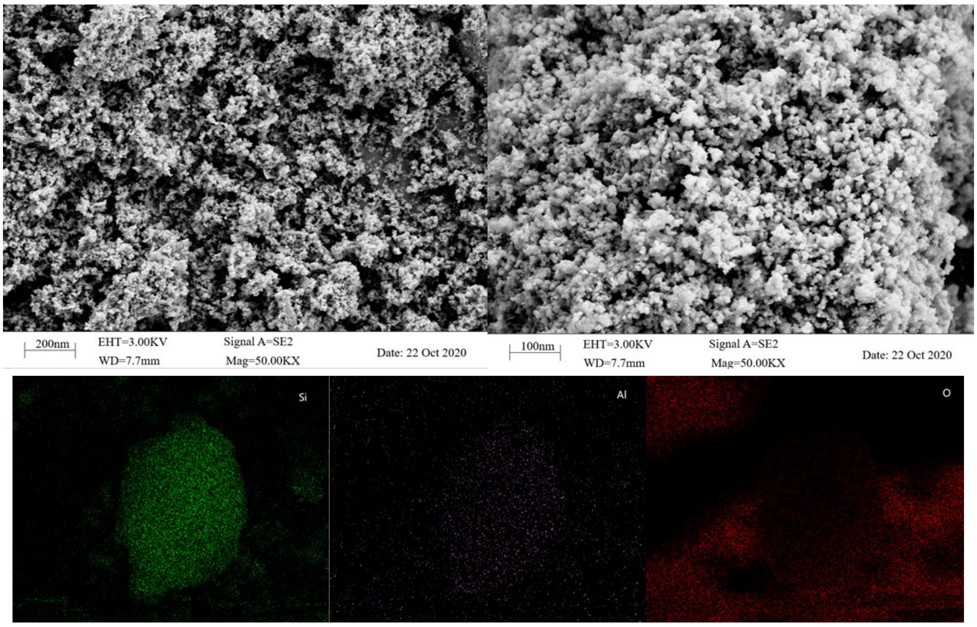

Fig. S2 Characterization of MCM-41 molecular sieve by SEM and mapping

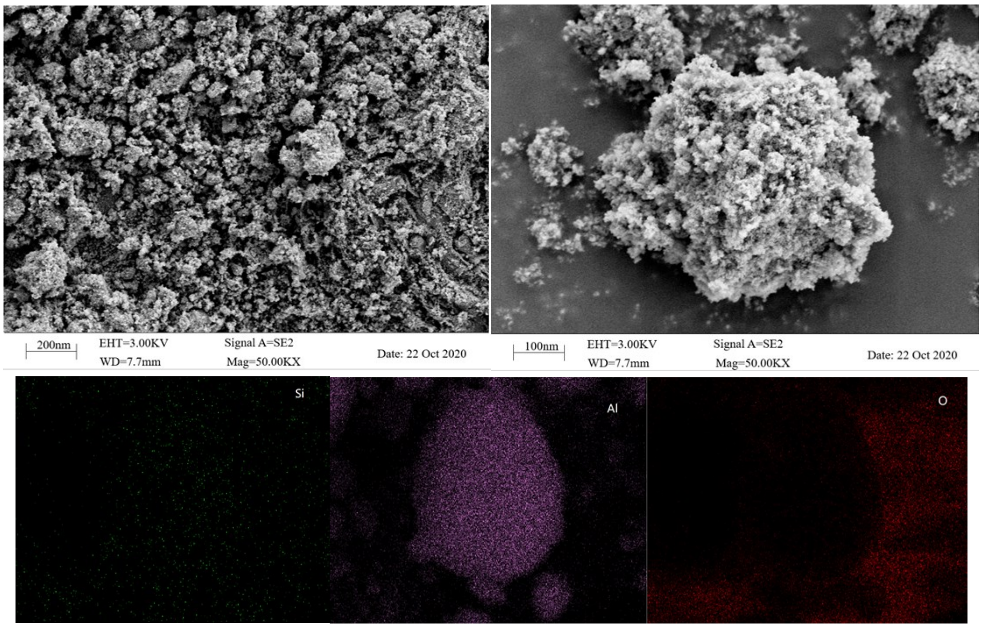

Fig. S3 Characterization of γ-Al_2O_3 molecular sieve by SEM and mapping

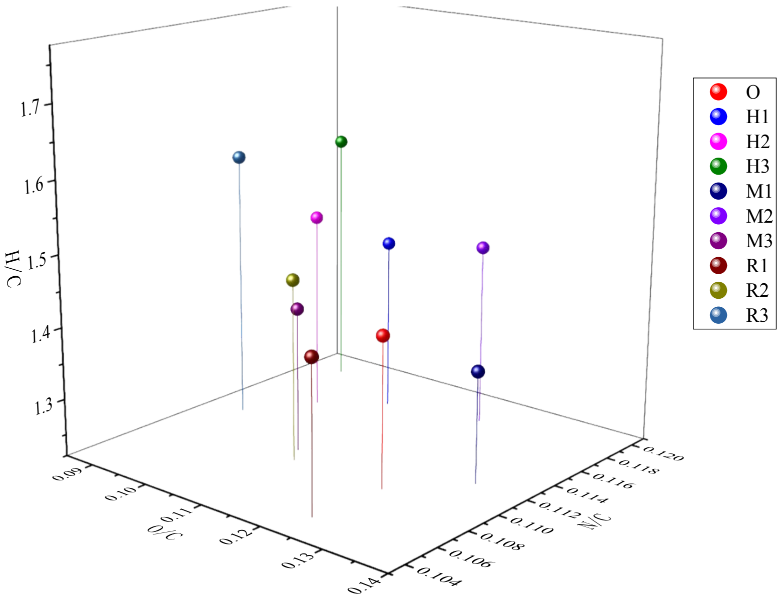

Fig. S4 Van Krevelen diagram of bio-oil obtained

Table S3 chemical composition of bio-oil

Residence time (min)	Compounds	O	H1	H2	H3	M1	M2	M3	R1	R2	R3
2.24	Methane, nitroso-	0.29	1.79	1.8	2.3	3.7	1.03	0.83	1.13	1.3	0.78
15.91	2,5-Pyrrolidinedione, 1-ethyl-	0.48	0.49	0.6	0.51	0.49	2.72	0.57	0.58	0.58	0.82
17.771	2-tetradecane, 5,7,13,15-tetramethyl-	-	0.21	1.06	1.76	0.12	0.43	0.96	0.33	0.56	0.7
18.835	2-hexadecene, 3,7,11,15-tetramethyl-	0.92	1.33	1.47	1.72	3.12	4.23	2.11	2.52	3.72	2.12
21.78	Indole	0.54	0.47	0.47	-	-	0.43	0.38	-	-	-
22.055	Pyrazine, 2-ethyl-3-methyl-	0.67	0.42	0.37	0.2	0.55	1.17	0.71	0.55	0.85	0.79
24.615	2,5-Pyrrolidinedione, 1-butyl-	-	-	0.6	0.3	-	0.46	0.58	-	-	-
28.89	2,4-Di-tert-butylphenol	1.11	0.62	0.79	0.96	0.68	0.82	-	0.59	0.65	0.68
35.6	Decanoic acid, 2-phenylethyl ester	0.71	0.54	0	1.13	0.63	-	-	0.61	0.58	0.62
38.04	Cyclo(L-prolyl-L-valine)	0.87	3.54	2.58	1.9	3.52	2.35	1.76	1.79	2.47	2.51
40.46	Pyrrolo[1,2-a]pyrazine-1,4-dione,hexahydro-3-(2-ethylpropyl)-	0.52	3.4	1.53	4.57	6.26	4.12	1.33	1.02	5.2	5.33
40.8	Dibutyl phthalate	0.52	0	0.59	0.49	0.74	0.75	0.69	1.48	0.59	0.55
41.37	n-He0adecanoic acid	2.67	0.82	0.93	1.22	0.68	0.53	0.66	1.36	1.16	1.08
41.925	He0adecanoic acid, ethyl ester	6.79	6.83	6.81	6.61	6.81	6.89	7.26	6.24	5.89	5.26
45.295	10(E),12(Z)-Conjugated linoleic acid	5.85	1.77	-	-	0.57	0.29	1.67	-	-	0.54
45.47	9,12-Octadecadienoic acid (Z,Z)-	4.53	4.61	6.3	4.93	3.06	2.15	4.7	7.69	6.73	10.45
46.06	Linoleic acid ethyl ester	28	36	26	23	35.39	31.77	36.48	33.69	31.87	32.36
46.5	Octadecanoic acid, ethyl ester	1.09	1.12	6.81	1.07	1.1	1.06	1.19	1.06	1.04	0.98
47.7	2,5-Piperazinedione, 3-benzyl-6-isopropyl-	0.75	1.94	3.69	1.14	2.78	3.54	2.11	2.1	3.4	2.32
47.76	9(E),11(E)-Conjugated linoleic acid, ethyl ester	2.5	-	2.62	2.69	-	2.68	1.1	-	2.29	2.08
49.1	Pyrrolo[1,2-a]pyrazine-1,4-dione, he0ahydro-3-(phenylmethyl)-	1.52	0.65	1.22	2.3	1.01	0.94	0.73	0.7	1.19	1
49.78	9,12-Octadecadienoic acid, methyl ester	1.83	1.91	2.05	2.18	1.78	2.12	2.16	1.85	1.85	1.97

50.075	13-Tetradece-11-yn-1-ol	0.89	0.87	2	2.27	0.73	0.99	1.1	0.78	0.73	0.78
51.615	Propanamide, 3-cyclopentyl-N-ethyl-	1.36	4.79	2.12	1.5	0.62	2.9	0.91	1.85	1.88	1.02
58.25	Eicosanoic acid, ethyl ester	0.59	0.66	0.65	0.58	1.29	0.42	0.62	0.63	1.03	0.69
60.825	3.alpha.,5-Cyclo-5.alpha.-ergosta-6,8(14).22t-triene	0.72	0.83	0.9	0.56	1.03	0.62	1	0.99	1.02	1.12
63.95	8(14),22-Ergostadienol	0.75	0.85	0.81	0.49	0.94	0.71	1.03	0.89	0.83	1.37
64.925	Neoergosterol	4.61	4.75	4.75	4.24	4.93	4.57	5.5	5.09	5.15	5.88
65.47	4-[9,10-Dihydrophenanthren-2-yl]-n-butanol	0.621	0.7	0.68	0.61	0.63	0.41	0.64	0.5	0.69	0.76
66.25	.gamma.-Sitosterol	3.11	3.41	3.6	3.46	3.86	3.19	3.99	3.95	4.07	4.02
66.9	Anthraergostatetraenol	1.65	1.85	1.75	2.02	1.87	1.75	2.13	2.01	1.3	2

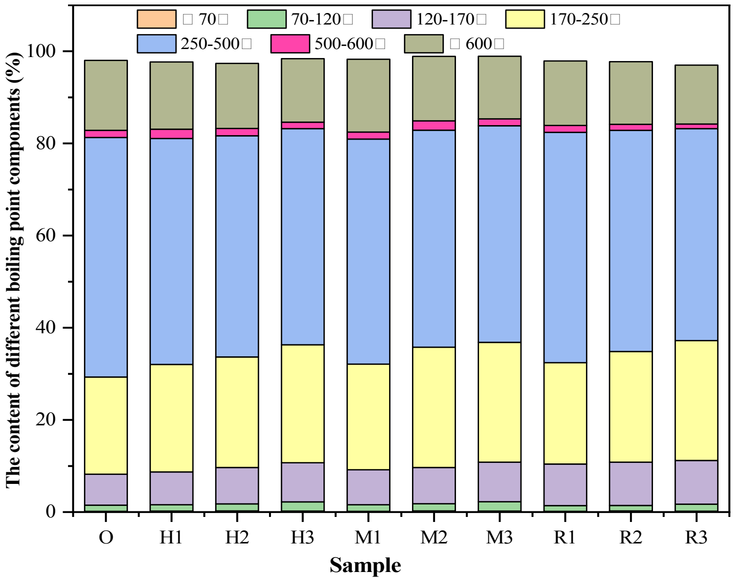

Fig.S5 Boiling range of fraction

Table S4 Percentage of H distribution from ^{1}H NMR spectra of the bio-oil

Sample	Chemical shift (Content w / %)				
	δ=0.5～1.5	δ=1.5～4.0	δ=3.0～4.4	δ=4.4～6.0	δ=6.0～8.5
O	61.42	22.33	1.33	8.8	6.12
H1	69.33	16.21	0.94	6.09	7.43
H2	75.14	10.12	0.85	5.12	8.77
H3	82.21	7.32	0.71	0.05	9.31
M1	62.35	19.05	1.02	11.57	6.01
M2	73.77	12.37	0.89	7.25	5.72
M3	79.69	10.33	0.80	3.63	5.55
R1	70.01	17.21	0.92	5.42	6.44
R2	77.31	12.98	0.79	1.94	6.98
R3	84.7	7.3	0.71	0.57	6.72
